# Post‐COVID‐19 Area Postrema Syndrome With SARS‐CoV‐2 in CSF: A Dual‐Case Report and Review of the Literature

**DOI:** 10.1002/iid3.70421

**Published:** 2026-04-06

**Authors:** Wan Zhu, Jing Qian, Min Peng, Yan Li, Jinghan Hu

**Affiliations:** ^1^ Department of Radiology, Affiliated Hospital, People's Hospital of Wenshan Prefecture Kunming University of Science and Technology Wenshan China; ^2^ Medical College of Kunming University of Science and Technology Kunming China; ^3^ Department of Respiratory Medicine The First People's Hospital of Yunnan Province Kunming China; ^4^ Department of Neurology, Affiliated Hospital, People's Hospital of Wenshan Prefecture Kunming University of Science and Technology Wenshan China; ^5^ The Affiliated Hospital of Kunming University of Science and Technology Kunming China

**Keywords:** aquaporin‐4 (AQP4‐IgG), area postrema syndrome, COVID‐19, neuromyelitis optica spectrum disorder (NMOSD), SARS‐COV‐2

## Abstract

**Background:**

Neuromyelitis optica spectrum disorder (NMOSD) is a rare autoimmune astrocytopathy characterized by inflammatory demyelinating lesions in the central nervous system. Area postrema syndrome (APS), marked by intractable nausea, vomiting, and hiccups, is a recognized but less common initial manifestation. Post‐infectious autoimmunity triggered by SARS‐CoV‐2 has been increasingly associated with NMOSD pathogenesis; however, the clinical significance of direct viral neuroinvasion and its relationship to divergent patient outcomes remains poorly understood.

**Methods:**

We report two female patients who developed isolated APS shortly after COVID‐19 infection. Both patients underwent comprehensive neurological evaluation, including brain and spinal magnetic resonance imaging (MRI), cerebrospinal fluid (CSF) analysis with metagenomic next‐generation sequencing (mNGS), and serological testing for aquaporin‐4 immunoglobulin G (AQP4‐IgG), myelin oligodendrocyte glycoprotein immunoglobulin G (MOG‐IgG), and glial fibrillary acidic protein immunoglobulin G (GFAP‐IgG) using cell‐based assays. Clinical outcomes were compared in the context of antibody serostatus and treatment strategies. A review of the relevant literature on post‐COVID NMOSD was also performed.

**Results:**

Both patients presented with intractable vomiting and hiccups following SARS‐CoV‐2 infection, and MRI demonstrated isolated T2/FLAIR hyperintense lesions in the dorsal medulla consistent with area postrema involvement. SARS‐CoV‐2 RNA sequences were detected in the CSF of both patients via mNGS, suggesting direct viral neuroinvasion or blood–brain barrier compromise. Despite similar initial presentations, their outcomes diverged dramatically. Patient 1 was AQP4‐IgG negative, responded well to immunotherapy with intravenous immunoglobulin and corticosteroids followed by mycophenolate mofetil maintenance, and remained relapse‐free at 12‐month follow‐up with significant lesion regression on MRI. Patient 2 was AQP4‐IgG positive in both serum and CSF, and despite acute treatment, experienced a fatal relapse 6 months later with longitudinally extensive transverse myelitis while on low‐dose prednisone monotherapy.

**Conclusions:**

Isolated APS may represent an important yet under‐recognized manifestation of post‐COVID‐19 autoimmune neuroinflammation. Detection of SARS‐CoV‐2 in CSF supports a role for direct viral neuroinvasion as a localized inflammatory stimulus. AQP4‐IgG serostatus serves as a critical prognostic determinant: seronegativity is associated with a benign, monophasic course, whereas seropositivity mandates prompt initiation of potent immunosuppressive therapy to prevent devastating relapses. Clinicians should maintain a high index of suspicion for NMOSD in patients with unexplained persistent vomiting following COVID‐19, and perform urgent neuroimaging and antibody testing for early risk stratification.

## Graphical abstract

This dual‐case report describes two patients with post‐COVID‐19 area postrema syndrome whose outcomes were dictated by their AQP4‐IgG serostatus, despite both having SARS‐CoV‐2 detected in their CSF. The stark contrast between the seronegative patient's recovery and the seropositive patient's fatal relapse highlights antibody testing as an urgent and critical step for prognosis and guiding immediate therapeutic strategy.

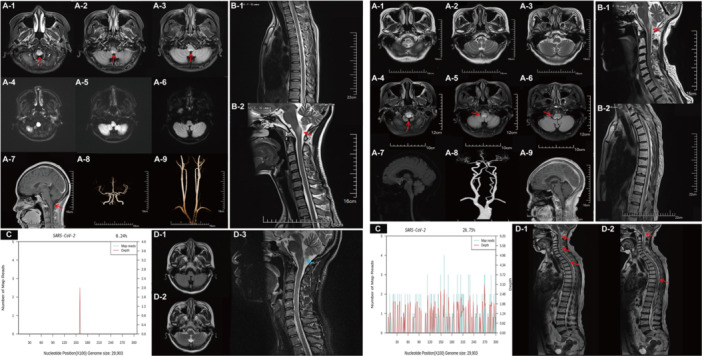

## 1 Introduction

Neuromyelitis optica spectrum disorder (NMOSD) is a relapsing autoimmune demyelinating disease of the central nervous system (CNS) primarily mediated by antibodies against aquaporin‐4 (AQP4) water channels on astrocytes. It classically affects the optic nerves and spinal cord, with core clinical manifestations, including optic neuritis, acute transverse myelitis, area postrema syndrome (APS), and acute brainstem syndrome. APS is defined by intractable nausea, vomiting, or hiccups not attributable to a gastrointestinal cause, and it often correlates with demyelinating lesions in the dorsal medulla (area postrema region) [1]. Indeed, APS is a hallmark feature of NMOSD and may be the initial presenting syndrome, especially in AQP4‐IgG positive patients. However, APS can easily be misdiagnosed as a GI or psychiatric disorder, leading to delays in recognizing underlying neuroinflammation [1, 2].

Viral infections are known environmental triggers for autoimmune demyelination, and 15%–35% of NMOSD cases are preceded by recent infections [3]. The novel coronavirus disease 2019 (COVID‐19), caused by SARS‐CoV‐2, has been associated with a wide range of post‐infectious neurological complications. SARS‐CoV‐2 can dysregulate the host immune system and induce a cascade of immune‐mediated CNS damage. Since the pandemic's onset, multiple case reports have linked COVID‐19 with CNS demyelinating events, including transverse myelitis, acute disseminated encephalomyelitis, and new‐onset NMOSD [4, 5, 6].

A systematic review by Harel et al. identified 15 patients who developed de novo NMOSD following COVID‐19 infection (median onset ~14 days post‐infection) [7]. Most reported cases were AQP4‐IgG seropositive and female (76%), consistent with typical NMOSD epidemiology. Proposed mechanisms for COVID‐19‐triggered autoimmunity include bystander lymphocyte activation, epitope spreading, molecular mimicry between viral and self‐antigens, and blood–brain barrier disruption allowing entry of autoantibodies [4, 5, 8]. This is supported by evidence of elevated cytokines (e.g., IL‐6) in COVID‐19 that can promote Th17 responses implicated in NMOSD immunopathology [9]. Another systematic review by Mirmosayyeb et al. (2022) similarly concluded that NMOSD is a conceivable, albeit rare, complication following COVID‐19 [10].

In this context, we report two cases of isolated area postrema syndrome following SARS‐CoV‐2 infection in female patients. Despite similar initial presentations, one case remained monophasic without detectable AQP4‐IgG, whereas the other was AQP4‐IgG positive and evolved into relapsing NMOSD. We describe their clinical course, diagnostic workup, and management, and discuss the pathophysiological hypotheses and implications for diagnosis and therapy. These cases underscore the importance of early recognition of APS in the post‐COVID period and the need to distinguish transient post‐infectious phenomena from the unmasking of a chronic autoimmune disease.

## 2 Case Description (Table 1)

**Table 1:** Comparative summary of two post‐COVID APS cases.

Feature	Patient 1	Patient 2
Age	32 years	69 years
Sex	Female	Female
Comorbidities	None	Hypertension, resected lung cancer
COVID‐19 severity	Mild	Mild (initially)
Latency to APS onset	2 weeks	10 days (from paresthesia onset)
APS symptoms	Intractable nausea, vomiting, hiccups	Vomiting, dizziness, ataxia, hiccups
Physical examination	Spontaneous vertical nystagmus	Left lower limb muscle strength grade 4, right lower limb muscle strength grade 4‐, ataxia.
Initial brain MRI findings	Isolated T2/FLAIR hyperintense lesion in dorsal medulla (area postrema)	Isolated T2/FLAIR hyperintense lesion in dorsal medulla (area postrema)
Initial spine MRI findings	Normal	Normal
CSF findings	5 lymphocytes/µL, protein 56 mg/dL, No OCBs	Normal cells and protein
CSF SARS‐CoV‐2 mNGS	2 sequences (0.24% abundance)	148 sequences (26.75% abundance)
AQP4‐IgG status	Negative (serum)	Positive (Serum Titer 1:100; CSF Titer 1:10)
MOG‐IgG status	Negative (serum)	Negative (serum)
Acute treatment	IV methylprednisolone, IVIG, nirmatrelvir/ritonavir	IV methylprednisolone, IVIG, nirmatrelvir/ritonavir
Maintenance therapy	Mycophenolate Mofetil Tablets (1 g/day)	Prednisone (10 mg/day)
Clinical course	Monophasic, complete resolution	Relapsing, progressive
Final outcome	Relapse‐free at 12 months	Deceased 8 months after initial presentation

### 2.1 Patient 1

A 32‐year‐old woman with no significant past medical history developed mild COVID‐19 symptoms on February 1, 2024, including a low‐grade fever, cough, and anosmia. Her infection was confirmed by a nasopharyngeal reverse transcription‐polymerase chain reaction (RT‐PCR) test for SARS‐CoV‐2. After 10 days of conservative management, her respiratory symptoms resolved completely. However, on Day 24 post‐symptom onset (14 days after the resolution of respiratory symptoms), she began experiencing daily episodes of intractable nausea, non‐bilious vomiting, and persistent hiccups. These symptoms occurred in the absence of any other neurological complaints, such as headache, dizziness, limb weakness, sensory disturbances, or visual changes. After 7 days of persistent vomiting led to dehydration, she was admitted to the hospital for further evaluation.

### 2.2 Investigations

**Figure 1:**
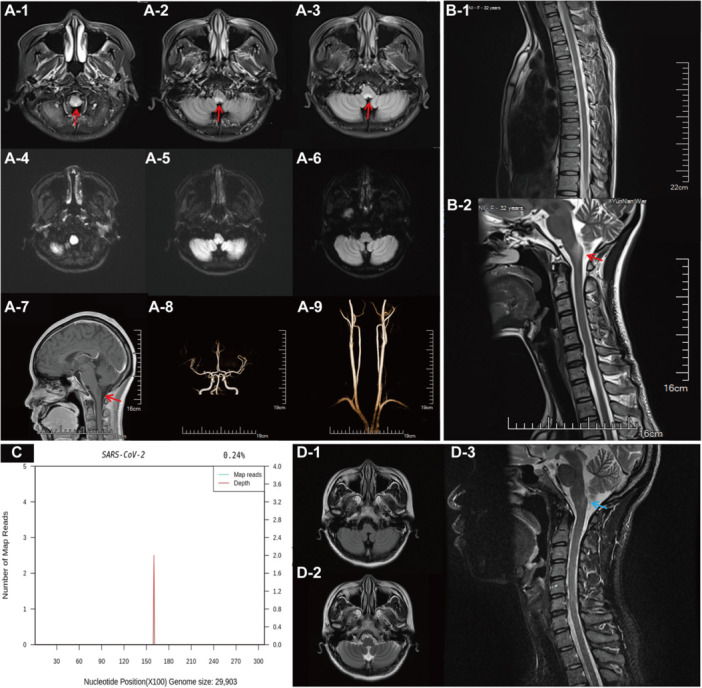
Patient 1's baseline craniospinal MRI on March 2, 2024, cerebrospinal fluid mNGS test results on March 14, 2024, and follow‐up craniospinal MRI on March 20, 2025. (A1–6) Brain MRI shows FLAIR hyperintensity (V‐sign) and slightly high DWI signal (arrows) posterior to the medulla‐spinal cord junction. (A7–9) Contrast‐enhanced brain MRI shows no enhancement, and head‐neck CTA reveals no abnormalities. (B1–2) Sagittal spinal MRI demonstrates isolated T2 hyperintensity posterior to the medulla‐spinal cord junction. (C) Cerebrospinal fluid mNGS on March 14, 2024, detected 2 SARS‐CoV‐2 RNA gene sequences with a relative abundance of 0.24%. (D1–3) Follow‐up craniospinal MRI 1 year after discharge shows reduced lesion size and signal softening posterior to the medulla‐spinal cord junction.

On admission, intermittent hiccups and vertical nystagmus were observed, with no other abnormalities in physical or neurological examinations. Cranial nerve function, muscle strength, sensation, and coordination were all normal. Initial laboratory tests (including blood biochemistry) and abdominal ultrasound showed no abnormalities. Standard antiemetic treatment was minimally effective. Given the persistent and unexplained symptoms following COVID‐19 infection, a neurogenic etiology was considered. Brain magnetic resonance imaging (MRI) revealed an isolated T2‐weighted fluid‐attenuated inversion recovery (FLAIR) hyperintense lesion in the dorsal medulla, localized to the area postrema at the floor of the fourth ventricle. Axial imaging showed a characteristic inverted “V” shape without gadolinium enhancement (Figure 1A1–A7). Full‐spine MRI was normal (Figure 1B1–B2,A8–A9). Brainstem visual evoked potentials indicated prolonged bilateral P100 latencies. Lumbar puncture cerebrospinal fluid (CSF) analysis showed mildly elevated protein (56 mg/dL), a lymphocyte count of 5/µL, and no oligoclonal bands. Notably, CSF metagenomic next‐generation sequencing (mNGS) detected two SARS‐CoV‐2 sequences with a relative abundance of 0.24% (Figure 1C). Serum and cerebrospinal fluid NMOSD‐related demyelination tests, including AQP4‐IgG, MOG‐IgG, and GFAP‐IgG (cell‐based assay), and antinuclear antibodies, were negative, with no other infectious or autoimmune markers detected.

Treatment and Outcome: Based on the characteristic clinical syndrome and MRI findings, a diagnosis of isolated APS associated with post‐COVID‐19 immune dysregulation was made. The patient was treated with a 5‐day course of nirmatrelvir/ritonavir, intravenous immunoglobulin (IVIG) at a dose of 0.4 g/kg/day for 5 days, and high‐dose intravenous methylprednisolone (1 g/day for 5 days), followed by oral prednisone tapering (starting at 60 mg/day, reduced by 10 mg every week). Her symptoms improved rapidly. Given the severity of her initial presentation, a decision was made to initiate maintenance therapy with Mycophenolate Mofetil Tablets (1 g/day) to mitigate any risk of future relapse. At her 12‐month follow‐up, she remained completely asymptomatic and relapse‐free on this treatment. A follow‐up MRI at that time showed significant regression of the medullary lesion, with no new lesions apparent (Figure 1D1–D3).

### 2.3 Final Diagnosis

Monophasic post‐infectious brainstem demyelinating syndrome (isolated APS).

### 2.4 Patient 2

A 69‐year‐old woman with a history of hypertension and stage IA lung adenocarcinoma treated with curative resection 3 years prior, with no evidence of recurrence on recent PET‐CT, presented to the hospital with dizziness, vomiting, and ataxia. Ten days prior to the onset of neurological symptoms, she developed fever and cough, and tested positive for SARS‐CoV‐2. Her neurological presentation began with plantar paresthesia. Upon presentation, she was febrile with a temperature of 39°C, and a SARS‐CoV‐2 PCR test was positive. An initial gastrointestinal evaluation, including an upper endoscopy and abdominal imaging, was unremarkable.

**Figure 2:**
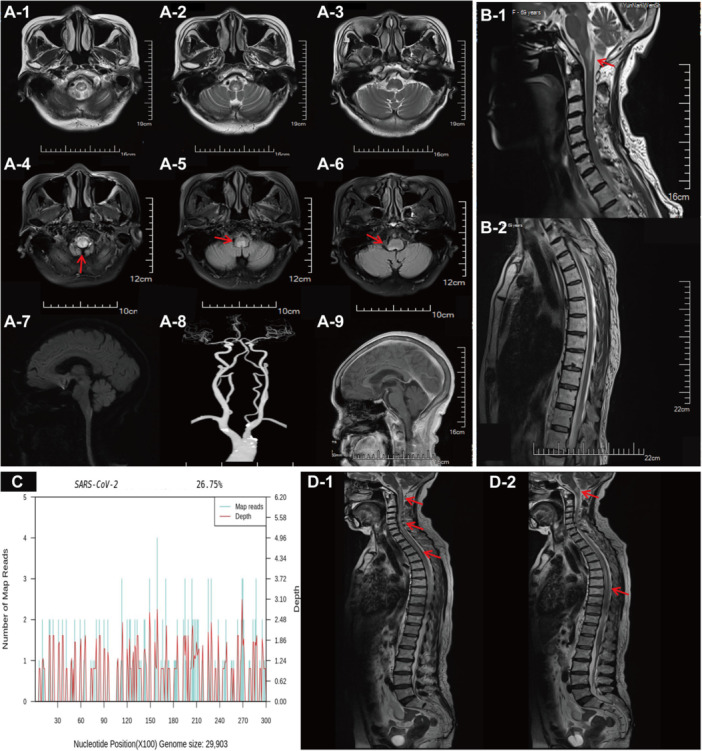
Patient 2's baseline craniospinal MRI on April 20, 2024, cerebrospinal fluid mNGS test results on May 2, 2024, and recurrent craniospinal MRI on October 17, 2024. (A1–6) Brain MRI shows T2/FLAIR hyperintensity involving the dorsal medulla at the level of the cervicomedullary junction (V‐sign, arrows). (A7–9) Brain DWI, head‐neck CTA, and contrast‐enhanced brain MRI show no abnormalities. (B1–2) Sagittal spinal MRI reveals isolated T2 hyperintensity posterior to the medulla‐spinal cord junction. (C) Cerebrospinal fluid mNGS on May 2, 2024, detected 148 SARS‐CoV‐2 RNA gene sequences with a relative abundance of 26.75%. (D1–2) Six months after discharge, the patient developed lower limb weakness and sphincter dysfunction, with follow‐up MRI showing longitudinally extensive transverse myelitis (LETM) extending from the medulla to the thoracic spinal cord.

Investigations: Neurological examination was notable for frequent hiccups, bilateral lower limb weakness, and gait ataxia, but no nystagmus. A brain MRI identified a single T2/FLAIR hyperintense lesion in the dorsal medulla, consistent with area postrema involvement. The lesion did not show gadolinium enhancement or signs of acute infarction. No other lesions were observed in the cerebrum, optic nerves, or cerebellum, and an initial spinal MRI showed no evidence of myelitis (Figure 2A1–A7,B1–B2). Serological testing for other rheumatologic and inflammatory markers, including ANA, anti‐dsDNA antibodies, ESR, and CRP, was normal. Screening for paraneoplastic antibodies (Hu, Yo, Ri, Ma2, and CV2) in serum and CSF was negative. Paraneoplastic‐related antibody tests in cerebrospinal fluid and blood were negative. CSF analysis revealed normal cell counts and protein levels. However, in stark contrast to Patient 1, testing for AQP4‐IgG (cell‐based assay, CBA) was positive in both the serum (titer 1:100) and the CSF (titer 1:10). Furthermore, CSF mNGS detected 148 SARS‐CoV‐2 sequences, corresponding to a relative abundance of 26.75% (Figure 2C).

Treatment and Outcome: The patient was diagnosed with AQP4‐positive NMOSD, likely triggered by her SARS‐CoV‐2 infection, presenting as APS. She was treated with a 5‐day course of nirmatrelvir/ritonavir, high‐dose corticosteroids, and IVIG. Her condition improved, and she was discharged with the ability to ambulate independently. For long‐term relapse prevention, she was prescribed a maintenance regimen of oral prednisone at 10 mg per day, with a planned duration of 1 year.

Relapse and Final Outcome: However, this low‐dose prednisone monotherapy proved insufficient to prevent relapse in this high‐risk phenotype. Six months after discharge, she experienced a severe, fatal relapse. A repeat MRI at that time revealed a new, longitudinally extensive transverse myelitis (LETM) lesion extending from the medulla down to the thoracic spinal cord (Figure 2D1–D2). She was aggressively retreated with IVIG and five sessions of plasma exchange. However, her neurological condition continued to decline, progressing to respiratory failure. She died 2 months after the relapse.

Final Diagnosis: AQP4‐IgG‐positive NMOSD, triggered by SARS‐CoV‐2 infection.

## 3 Discussion

This report presents a compelling juxtaposition of two patients with identical presentations of isolated area postrema syndrome (APS) temporally associated with SARS‐CoV‐2 infection, whose clinical trajectories diverged dramatically based on their AQP4‐IgG serostatus. This direct, side‐by‐side comparison provides significant didactic value, illustrating how a common infectious trigger can initiate two distinct neuroinflammatory pathways: a self‐limiting, monophasic illness vs. a fulminant, relapsing autoimmune disease.

A pivotal finding in this study is the detection of SARS‐CoV‐2 RNA sequences in the cerebrospinal fluid (CSF) of both patients via metagenomic next‐generation sequencing (mNGS). The link between infection and autoimmunity is likely multifactorial. Molecular mimicry, where structural similarities between SARS‐CoV‐2 antigens and self‐proteins like AQP4 trigger cross‐reactive antibodies, is a leading hypothesis [11]. Another key mechanism is bystander activation, wherein the massive infection‐driven cytokine release lowers the threshold for autoreactive lymphocytes [4, 5]. SARS‐CoV‐2 is known to induce a systemic inflammatory state with elevated IL‐6 [9, 12], a cytokine strongly implicated in NMOSD immunopathology by promoting plasmablast differentiation and AQP4‐IgG production [12]. Finally, the infection may unmask a latent autoimmune predisposition, precipitating the first clinical attack in a patient with subclinical autoimmunity [7]. In our Case 2, it is highly plausible that the infection‐induced cytokine surge tipped a subclinical autoimmune state into overt, catastrophic NMOSD. In contrast, Case 1 likely represents a transient, para‐infectious response that resolved without establishing chronic autoimmunity.

Detection of SARS‐CoV‐2 RNA in cerebrospinal fluid (CSF) is not part of standard diagnostic workflows for most post‐COVID neurological syndromes, and positive results require cautious interpretation. However, case reports support its potential clinical relevance in selected presentations. Moriguchi et al. reported meningitis/encephalitis in which SARS‐CoV‐2 RNA was detected in CSF despite a negative nasopharyngeal swab, suggesting that CSF positivity, although uncommon, may occur in clinically meaningful CNS involvement [13]. In our cases, detection of SARS‐CoV‐2 sequences by metagenomic next‐generation sequencing (mNGS) may reflect direct neuroinvasion in a susceptible anatomical region and/or blood–brain barrier disruption with viral RNA entry into CSF. Importantly, detection of viral RNA does not necessarily indicate active replication in the CNS, and may represent low‐level or fragmented viral material or technical contamination. Therefore, CSF SARS‐CoV‐2 positivity should be interpreted as supportive evidence that strengthens biological plausibility when aligned with the clinical syndrome, neuroimaging, and exclusion of alternative infectious etiologies, rather than as a standalone diagnostic criterion.

The starkly divergent outcomes in our two patients underscore the critical need to understand the prognostic factors in post‐COVID‐19 neuroinflammation. Our findings are contextualized by an accumulating body of literature, a selection of which is summarized in Table 2. AQP4‐IgG positive NMOSD is the most frequently reported severe outcome, often presenting with classic features like LETM or APS and following a relapsing course, as seen in our Patient 2 and cases by Batum et al. [4], Correa et al. [5], Ghosh et al. [6], and Shaw et al. [15]. Some reports even suggest a broader post‐infectious autoimmune response, with NMOSD co‐occurring with conditions like acute myositis [14]. Conversely, a distinct subgroup of patients, like our Patient 1, experiences a monophasic, seronegative illness that resolves fully, as documented by Rafique et al. [3] and de Ruijter et al. [16]. Furthermore, our review highlights the critical importance of considering MOG antibody‐associated disease (MOGAD) as a key differential diagnosis, as cases of MOG‐IgG positive APS post‐COVID have been reported [8]. A systematic review by Mirmosayyeb et al. confirmed that the median latency from infection to neurologic onset is approximately 2 weeks, consistent with a post‐infectious process [10]. This comprehensive view reinforces our central thesis: while the initial trigger and presentation can be similar, antibody status is the paramount factor determining the underlying pathology, prognosis, and long‐term management.

**Table 2:** Comprehensive review of representative cases of post‐COVID‐19 demyelinating syndromes presenting with area postrema syndrome (APS) or NMOSD.

Case (author, year)	Patient (age/sex)	Presenting syndrome(s)	AQP4‐IgG serum/CSF	Anti‐GFAP serum/CSF	MOG‐IgG serum/CSF	CSF SARS‐CoV‐2 RNA	Outcome	Key features & comments
Ghosh et al. (2020) [6]	20/M	APS, evolving to LETM	Positive	Not reported	Negative	Negative	Improved relapse‐free at 4 months	First reported AQP4 + NMOSD post‐COVID, presented with APS.
Correa et al. (2021) [5]	51/F	LETM, encephalitis	Positive	Not reported	Not reported	Negative	Improved with treatment	Raised “cause or coincidence” question; required immunosuppression.
Rafique et al. (2021) [3]	7.5/F	APS, optic neuritis, myelitis	Negative	Not reported	Negative	Not reported	Gradual improvement	Pediatric seronegative case.
Chen et al. (2024) [2]	26/F	APS, evolving to LETM	Positive	Not reported	Negative	Not reported	Severe, partial recovery	Emphasized diagnostic delay due to misdiagnosis as GI/psychiatric issue.
Batum et al. (2022) [4]	50/F	LETM	Positive	Not reported	Negative	Not reported	Poor recovery from single event	Classic NMOSD presentation triggered by COVID‐19.
Barone et al. (2021) [14]	35/M	Optic neuritis, myositis, APS	Positive	Not reported	Not reported	Not reported	Relapsing	Concurrent myositis suggests a broader post‐COVID autoimmune response.
Feizi et al. (2022) [8]	41/M	Encephalomyelitis, optic neuritis	Negative	Not reported	Positive	Negative	Relapsing (MOGAD)	This paper's own case was MOGAD, not NMOSD.
Shaw et al. (2020) [15]	70/M	Optic neuritis, LETM	Positive	Not reported	Not reported	Negative	Fatal	Early case report highlighting severe presentation and fatal outcome.
de Ruijter et al. (2020) [16]	15/M	Bilateral optic neuritis	Negative	Not reported	Positive	Not reported	Monophasic, recovery	An early report of MOGAD, not seronegative NMOSD.
Li and Teng (2025) [17]	59/F	APS, meningoencephalomyelitis	Negative	Positive	Negative	Not reported	Relapsing	Linear enhancement of brainstem surface; CSF pleocytosis (54/µL).
Li and Teng (2025) [17]	71/M	APS, psychiatric symptoms	Negative	Positive	Negative	Not reported	Relapsing	CSF pleocytosis (68/µL); respiratory failure.
Li and Teng (2025) [17]	57/M	APS, Horner syndrome	Negative	Positive	Negative	Not reported	Relapsing	Long duration of APS (40 days); CSF pleocytosis (70/µL).
Li and Teng (2025) [17]	12/F	APS, meningoencephalomyelitis	Positive	Positive	Negative	Not reported	Relapsing	Double positive (AQP4 + GFAP); extensive spinal cord lesions.
Present, Case 1	32/F	Isolated APS	Negative	Negative	Negative	Positive	Monophasic, full recovery	SARS‐CoV‐2 RNA in CSF; no relapse at 12 months.
Present, Case 2	69/F	APS, ataxia	Positive serum (1:100)	Negative	Negative	Positive	Relapsing, fatal	SARS‐CoV‐2 RNA in CSF; fatal relapse with LETM occurred due to insufficient maintenance therapy (low‐dose prednisone monotherapy).

Given the seronegativity in Patient 1, consideration of alternative etiologies, such as autoimmune glial fibrillary acidic protein (GFAP) astrocytopathy, is essential. Recent work by Li et al. [17] has characterized APS in GFAP‐IgG positive patients, noting that while less common than in NMOSD, it presents with distinct features. In their cohort, APS was the initial manifestation in all reported cases, and hiccups were highly prevalent (81%). However, a key distinguishing feature of GFAP‐associated APS is the presence of marked inflammation. Li and Teng reported a significantly higher frequency of leptomeningeal enhancement (61.9% vs. 5% in NMOSD) and elevated CSF white blood cell counts (median 120 cells/mm³). In contrast, our Patient 1 exhibited no leptomeningeal enhancement on MRI and had a low CSF lymphocyte count (5 cells/µL). This clinical and radiological profile aligns more closely with a post‐viral inflammatory syndrome than with the hyper‐inflammatory phenotype typical of autoimmune GFAP astrocytopathy. Nevertheless, clinicians should remain vigilant for GFAP‐IgG in seronegative APS cases, particularly when leptomeningeal enhancement is present.

These findings serve as a crucial clinical alert. APS is notoriously misdiagnosed, often attributed to non‐neurological causes, leading to critical delays in treatment [18]. As recent reports highlight, patients with post‐COVID APS can suffer diagnostic delays involving multiple GI evaluations or even psychiatric referrals before an MRI finally reveals the causal medullary lesion [1, 2]. Our cases and the literature review reinforce that intractable vomiting in a post‐COVID setting is a neurological red flag warranting urgent brain MRI and mandatory serological testing for both AQP4‐IgG and MOG‐IgG using high‐sensitivity cell‐based assays, as recommended by international consensus criteria [19].

These divergent pathways profoundly impact therapeutic strategies, and our cases serve as a critical lesson in applying this knowledge. The tragic outcome of Patient 2 underscores this urgency. While she received appropriate acute therapy with high‐dose corticosteroids and IVIG, the critical failure occurred in the choice of long‐term relapse prevention. A maintenance regimen of low‐dose prednisone monotherapy (10 mg/day) was initiated. This strategy, which deviates significantly from modern evidence‐based guidelines, proved insufficient to prevent relapse in this high‐risk phenotype. This case underscores that AQP4‐IgG positivity indicates a high risk of recurrence, supporting the consideration of potent immunosuppressive therapy. Indeed, managing such rare diseases during the COVID‐19 pandemic presented unique challenges, and ensuring adherence to optimal treatment protocols was a documented concern [20]. Modern international guidelines strongly advocate for a swift transition to potent therapies like B‐cell depletion (e.g., rituximab), complement inhibitors (e.g., eculizumab), or IL‐6 receptor blockers (e.g., satralizumab) precisely because they are necessary to prevent such devastating relapses [21, 22].

In stark contrast, the favorable outcome in Patient 1 was secured by the prompt initiation of an appropriate maintenance therapy with mycophenolate mofetil. Although long‐term immunosuppression is often debated in seronegative cases, the severity of her initial presentation warranted a proactive strategy, which was ultimately successful. Therefore, this tale of two outcomes illustrates a crucial clinical lesson: AQP4‐IgG positivity is not merely a label but a mandate for immediate and aggressive, evidence‐based immunosuppression. The divergence in their paths may reflect both the inherent biology of their disease and the profound impact of the subsequent therapeutic choices—in this case, the choice of an inadequate treatment vs. the choice to treat appropriately.

Several limitations of the present study should be acknowledged. As a dual‐case report, the findings cannot be generalized, and the divergent outcomes are illustrative rather than statistically significant. While the temporal association is compelling, this study cannot definitively establish causality. The pathophysiological mechanisms discussed remain inferential, and the direct role of CSF‐detected SARS‐CoV‐2 RNA in triggering the specific immune responses observed requires further investigation.

## 4 Conclusion

Our study reports that isolated area postrema syndrome is a rare yet severe neurological complication following SARS‐CoV‐2 infection. The detection of viral sequences in cerebrospinal fluid may serve as a localized inflammatory stimulus, further supporting the view of SARS‐CoV‐2 as a trigger for autoimmune responses in the central nervous system. AQP4‐IgG serological testing serves as a critical tool to differentiate these two distinct clinical pathways, thereby determining prognosis and guiding long‐term treatment strategies. AQP4 negativity suggests a benign, self‐limiting disease course, whereas AQP4 positivity indicates a high risk of recurrence, supporting the consideration of potent immunosuppressive therapy. These findings may offer broader insights into virus‐induced autoimmune mechanisms and provide a reference for future diagnosis and treatment of such complex cases.

## 5 Patient Perspective

The family of Patient 1 is satisfied with her diagnosis and successful treatment. The family of Patient 2, while grieving their loss, expressed understanding of the disease's aggressive nature and consented to share this case to help prevent similar outcomes in other patients.

## Author Contributions

**Wan Zhu:** conceptualization, data curation, writing – original draft, writing – review and editing. **Jing Qian:** conceptualization, data curation, writing – original draft, writing – review and editing. **Min Peng:** software, validation, writing – original draft. **Yan Li:** formal analysis, project administration, writing – review and editing. **Jinghan Hu:** conceptualization, data curation, formal analysis, writing – original draft, writing – review and editing.

## Ethics Statement

The studies involving humans were approved by the Ethics Committee of People's Hospital of Wenshan Prefecture. The studies were conducted in accordance with the local legislation and institutional requirements. The participants provided their written informed consent to participate in this study. Written informed consent was obtained from the individual (s) for the publication of any potentially identifiable images or data included in this article.

## Data Availability

The original data that support the findings of this study are included in the article and its supplementary materials. Further inquiries or requests for specific data can be directed to the corresponding author; however, some raw clinical data may be restricted to protect patient privacy.
